# A Clinical Case of a Celiac Crisis in an Adult With Type 1 Diabetes and Neurological Symptoms

**DOI:** 10.7759/cureus.35696

**Published:** 2023-03-02

**Authors:** Stanislav Kravchuk, Viktor Mishchanchuk, Marko Kozyk, Kateryna Strubchevska

**Affiliations:** 1 Internal Medicine, Novovolynsk Central City Hospital, Novovolynsk, UKR; 2 Infectious Diseases, Chortkiv Central City Hospital, Chortkiv, UKR; 3 Internal Medicine, Beaumont Hospital, Royal Oak, USA

**Keywords:** tissue transglutaminase, hypokalemia, gluten free diet, celiac disease, celiac crisis

## Abstract

We are reporting a case of a severe variant of celiac disease (celiac crisis) in an otherwise healthy 34-year-old woman presenting a history of weight loss, and neurological and metabolic disorders. After starting a gluten-free diet, the patient's condition improved significantly, and ascites and hydrothorax disappeared. The celiac crisis remains a rare manifestation of celiac disease among the adult population, however, a gluten-free diet should be considered in patients with marked metabolic disturbances even without significant osmotic diarrhea.

## Introduction

Celiac disease is one of the most common immune-mediated diseases of the small intestine affecting genetically susceptible individuals worldwide. It is triggered by the ingestion of gluten and other related proteins. The prevalence of the celiac disease has been estimated to approximate 0.5%-1% in different parts of the world [1,2]. It is manifested by a heterogeneous clinical picture, but typical symptoms include chronic diarrhea and signs of malabsorption. In some patients, celiac disease is accompanied by an acute onset that often requires hospitalization. Celiac disease is mostly found in children under two years of age [3]. Due to the relatively rare manifestation of this disease among the adult population, it is not easy or common for doctors to recognize the characteristic clinical picture of celiac disease [4].

## Case presentation

A 34-year-old female patient was hospitalized with complaints of impaired gait, severe general weakness, swelling of the lower extremities, and weight loss of more than 10 kg over the past two months. Her past medical history included type 1 diabetes mellitus. The patient denied fever, rash, catarrhal symptoms, and gastrointestinal symptoms. She reported being ill for the previous six months when edema of the lower extremities developed acutely. Later, due to psychological stress (the death of the brother), the above-mentioned symptoms significantly increased, the patient noted a sharp weight loss (by 7 kg within a week) while maintaining her appetite as well as normal caloric intake. The patient denied having viral hepatitis, HIV, or tuberculosis. She did not undergo medical examinations before the onset of the disease. She has not traveled abroad recently. Her family history is unremarkable. The patient denied having any sick contacts.

On examination, her neurological status was significant for flaccid paraparesis with predominant weakness of proximal muscles, absence of deep tendon reflexes in the lower extremities, and sensory ataxia. The patient's weight was 40 kg. Her body mass index (BMI) was 14.7 kg/m^2^. The skin was dry and pale with a yellowish tint. The patient had severe trophic changes: hair loss, lack of pubic and axillary hair, layering of nails, and tooth loss. There was no lymphadenopathy. Mucous membranes were pale with aphthous stomatitis. There was swelling of the lower extremities and the anterior abdominal wall. The patient's vital signs were within normal limits, with the exception of tachycardia of up to 110 beats per minute. During auscultation, a 3/6 systolic murmur was heard at the apex. The abdomen was distended, and tense with marked subcutaneous veins. She had a liver edge, palpable 4 cm below the costal margin.

**Figure 1:**
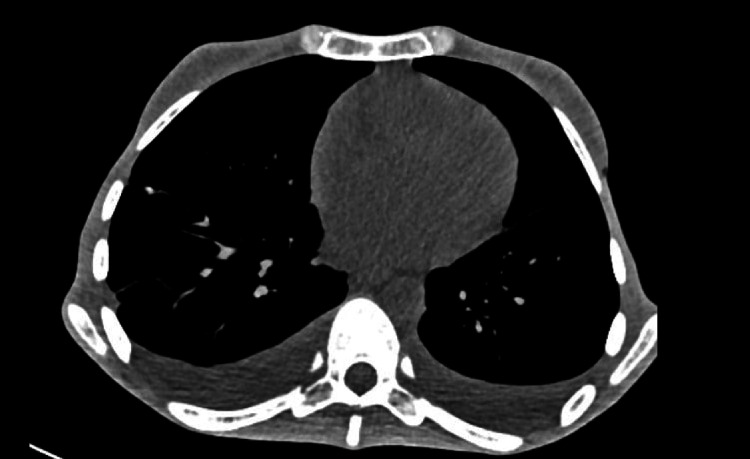
A CT scan of the abdominal organs with bilateral hydrothorax CT: computed tomography

Initial laboratory studies revealed serious electrolyte disturbances, elevated levels of liver enzymes, hyperglycemia, and low levels of plasma iron and albumin (Table 1). A computed tomography (CT) scan of the chest revealed bilateral hydrothorax and mild ascites (Figure 1).

**Table 1:** Laboratory studies carried out on admission

Blood Study	Normal Range	Results
Leukocytes	4-10.5 x 10^3^/uL	2.2 x 10^3^/uL
Neutrophils		43.9%
Hemoglobin	11.5-16.5 g/dL	8.7 g/dL
Platelets	150-450 x 10^3^/uL	109 x 10^3^/uL
Alanine transaminase	4-36 U/L	87.3 U/L
Aspartate aminotransferase	8-33 U/L	75.5 U/L
Alkaline phosphatase	35-104 U/L	177 U/L
Total bilirubin	<1 mg/dL	0.02 mg/dL
Gamma-glutamyl transferase	5-36 U/L	114.5 U/L
Total proteins	6.4-8.3 g/dL	2.6 g/dL
Albumin	3.4-4.8 g/dL	1.7 g/dL
Creatinine	0.5-0.9 mg/dL	0.7 mg/dL
Sodium	136-145 mEq/L	110.2 mEq/L
Potassium	3.5-5.1 mEq/L	2.9 mEq/L
Chloride	96-106 mEq/L	74.0 mEq/L
C-reactive protein	<5 mg/L	6.2 mg/L
Procalcitonin	<0.5 ng/mL	0.01 ng/mL
Glucose	70-99 mg/dL	302 mg/dL
Iron	60-170 mcg/dL	11.3 mcg/dL

**Figure 2:**
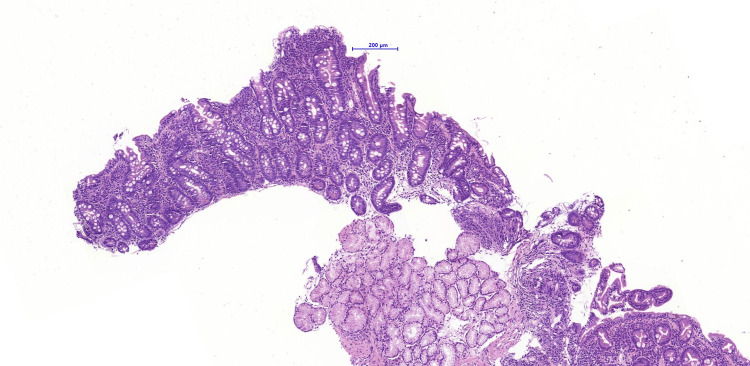
Duodenal biopsy showing villous atrophy, stain hematoxylin and eosin

Further diagnostic studies revealed elevated titers of immunoglobulin A (IgA) antibodies to tTGA (tissue transglutaminase) - 11.32 (normal: less than 1), with normal levels of the total level of IgA. The patient underwent upper endoscopy with a duodenal biopsy, which revealed atrophy of intestinal villi, an increased level of lymphohistioplasmacytic infiltration with a small number of neutrophils in the stroma of the lamina propria. The level of intraepithelial lymphocytosis was increased (60-70 lymphocytes per 100 enterocytes) (Figure 2).

The patient was started on a gluten-free diet. She also received albumin infusions, intravenous iron preparations (500 mg in total), and vitamin supplementation. Electrolyte status and hyperglycemia were corrected.

In one week, the patient's condition improved significantly. At discharge, there were no neurological deficits, edema, ascites, and hydrothorax.

## Discussion

Celiac crisis is a rare but potentially life-threatening clinical form of celiac disease that has been reported in both children and adults [3]. This condition was first described by Di Sant 'Agnese in 1953 in a case series of 35 children with a mortality rate of 9% [3,4]. Usually, cramping in severe celiac disease is accompanied by intense profuse diarrhea, severe dehydration, hemodynamic instability, as well as profound electrolyte and metabolic disturbances [3]. However, in this particular case, this condition was not accompanied by diarrhea. Unfortunately, the celiac crisis in adults is poorly documented in the medical literature [5,6]. To the best of our knowledge, there are only 40 described clinical cases and one systematic review. Despite the fact that this pathological condition has been known to the community for almost 70 years, it was only in 2010 that Jamma and his colleagues proposed criteria for defining a crisis in celiac disease [6]. To establish the condition an acute onset or rapid progression of gastrointestinal symptoms attributable to a celiac disease requiring hospitalization and/or parenteral nutrition should be present together with at least two definitive signs of malnutrition, dehydration, or electrolyte disturbance (Table 2) [6].

**Table 2:** Proposed definition and criteria for defining a crisis in celiac disease according to Jamma et al. [6]

Acute onset or rapid progression of gastrointestinal symptoms attributable to a celiac disease requiring hospitalization and/or parenteral nutrition along with at least two of the following:
Severe dehydration
Neurologic dysfunction
Increase in creatinine >2.0 g/dL
Metabolic acidosis: pH <7.35
Hypoproteinemia (Albumin <3.0 g/dL)
Abnormal electrolytes including hyper/hyponatremia, hypocalcemia, hypokalemia, or hypomagnesemia
Weight loss >5 kg

Our case meets five of the proposed criteria; weight loss, severe dehydration, severe hypokalemia/hyponatremia, neurological symptoms, with hypoalbuminemia.

## Conclusions

Celiac crisis is a limited, but critical manifestation of celiac disease. Due to the small number of documented cases among the adult population, this condition is rarely diagnosed. Although profuse diarrhea is one of the most common manifestations of celiac disease, it may not always be present, as our case describes. Hypokalemia is a frequent manifestation of electrolyte disturbances. Treatment consists of supportive therapy and a strict gluten-free diet. For patients with severe forms of celiac disease, correction of macro- and micronutrients should also be carried out.
